# Correlated Variability in the Breathing Pattern and End-Expiratory Lung Volumes in Conscious Humans

**DOI:** 10.1371/journal.pone.0116317

**Published:** 2015-03-24

**Authors:** Raffaele L. Dellaca, Andrea Aliverti, Antonella Lo Mauro, Kenneth R. Lutchen, Antonio Pedotti, Bela Suki

**Affiliations:** 1 Dipartimento di Elettronica, Informatica e Bioingegneria—DEIB, Politecnico di Milano University, Milano, Italy; 2 Biomedical Engineering Department, Boston University, Boston, Massachusetts, United States of America; New York University School of Medicine, UNITED STATES

## Abstract

In order to characterize the variability and correlation properties of spontaneous breathing in humans, the breathing pattern of 16 seated healthy subjects was studied during 40 min of quiet breathing using opto-electronic plethysmography, a contactless technology that measures total and compartmental chest wall volumes without interfering with the subjects breathing. From these signals, tidal volume (V_T_), respiratory time (T_TOT_) and the other breathing pattern parameters were computed breath-by-breath together with the end-expiratory total and compartmental (pulmonary rib cage and abdomen) chest wall volume changes. The correlation properties of these variables were quantified by detrended fluctuation analysis, computing the scaling exponentα. V_T_, T_TOT_ and the other breathing pattern variables showed α values between 0.60 (for minute ventilation) to 0.71 (for respiratory rate), all significantly lower than the ones obtained for end-expiratory volumes, that ranged between 1.05 (for rib cage) and 1.13 (for abdomen) with no significant differences between compartments. The much stronger long-range correlations of the end expiratory volumes were interpreted by a neuromechanical network model consisting of five neuron groups in the brain respiratory center coupled with the mechanical properties of the respiratory system modeled as a simple Kelvin body. The model-based α for V_T_ is 0.57, similar to the experimental data. While the α for T_TOT_ was slightly lower than the experimental values, the model correctly predicted α for end-expiratory lung volumes (1.045). In conclusion, we propose that the correlations in the timing and amplitude of the physiological variables originate from the brain with the exception of end-expiratory lung volume, which shows the strongest correlations largely due to the contribution of the viscoelastic properties of the tissues. This cycle-by-cycle variability may have a significant impact on the functioning of adherent cells in the respiratory system.

## Introduction

Most studies on physiological control systems have been based on measures of the average output over some period of time, and little attention has been paid to the mechanisms that determine how these control systems regulate their output in order to maintain a stable homeostatic internal environment. External fluctuations acting on the feedback loops within the control system often result in significant variabilities of the output, and it is becoming evident that variabilities carry useful information on the underlying structure and/or functioning of control systems [1].

One of the key life-support control systems of the body is the respiratory system. Many physiological variables associated with breathing such as tidal volume (V_T_) or respiratory rate exhibit significant breath-to-breath variabilities [2–5]. While some studies correlated variability and irregularity in breathing pattern with the presence of obstructive lung disease [6] or the maturation in preterm infants [4,7], the origin of the variabilities is not well understood even in normal subjects.

In an effort to account for the correlated variability in V_T_ observed in babies, Cernelc et al. [4] proposed to add noise to the neural network model of the brain respiratory oscillator put forth by Botros and Bruce [8]. Their modeling results showed that the phrenic output of the model generated a cyclic pattern with breath-to-breath variations that mimicked the correlation properties of V_T_ [4]. However, the respiratory system is composed of several mechanical structures such as the lung and the chest wall and its abdominal and rib cage compartments. It is not known whether the centrally controlled signal from the phrenic nerve results in different variabilities of these compartments. Additionally, the role of the passive mechanical properties of the lung and chest wall in the breath-to-breath variations of respiratory parameters has not been determined.

In this study, we characterised the variability of resting breathing pattern in healthy adult humans by using a technique called Optoelectronic Plethysmography (OEP)[9–11] that is capable of measuring all ventilatory parameters, including long-term changes in end-expiratory volume (EEV) changes [12] on a breath-by-breath basis both for the total respiratory system and as well as for the different chest wall compartments such as pulmonary rib cage (RC,p), abdominal rib cage (RC,a) and abdomen (AB). This technique provides an ideal opportunity to assess variabilities of the total and compartmental ventilatory parameters as it does not require a mouthpiece and/or noseclips and it does not add dead space or mechanical load to the subjects. We thus quantified the variability and the long-range correlation properties in the breathing pattern and chest wall volumes using the detrended fluctuation analysis (DFA) [13] technique which can detect intrinsic correlation properties of complex time series. Moreover, to interpret our results, we also developed a simple model of the chest wall and the lung driven mechanically by the respiratory muscles which in turn receive input from the respiratory rhythm generator in the brain.

## Methods

### Experimental study

**Fig 1:**
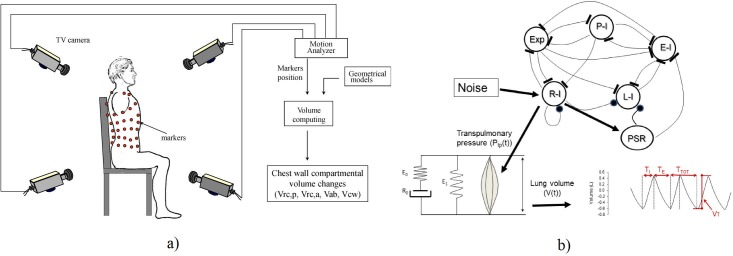
A): Experimental set-up for continuous recording of total and compartmental chest wall volumes with the patient in the seated position. A specially-designed chair allowed the measurement of the three-dimensional position of the passive optical markers for OEP placed on the back of the subject even if the subject was laid on the seatback. **B**): Schematic representation of the model. The brain respiratory rhythm generator includes five neuron groups, expiratory (Exp), early inspiratory (E-I), ramp-inspiratory (R-I), late-inspiratory (L-I) and post-inspiratory (P-I). Each group receives inhibitory signals (represented by a thick line at the end of the link between two groups) and the L-I group receives an excitatory input (filled circle). Note that the R-I group includes self-excitation. Pulmonary stretch receptors (PSR) are represented as an additional group of neurons receiving inputs from the R-I and sending outputs to the L-I and E-I groups. To mimic the observed physiological fluctuations in respiratory parameters such as tidal volume (V_T_) and respiratory times (T_TOT_ = T_I_+T_E_), the R-I group also receives a uniformly distributed neural noise and its output drives the respiratory muscles which, in turn, act on the passive structures of the respiratory system (lung and chest wall) represented by a viscoelastic Kelvin body to develop the tidal breathing. (See text for further explanation).

Sixteen young healthy subjects, 9 males and 7 females, age 28.3±4.6 years were studied while resting on a specially designed chair with their arm supported by armrests during a period of 35–40 minutes. The population was selected by considering healthy young subjects in order to characterize the variability pattern in a rather homogeneous group. Patients were studied in a seated position as we wanted to resume the standard posture in which FRC is normally evaluated. Chest wall volume (Vcw) was measured plethysmographically by OEP (Fig. 1A), a technique extensively validated in several conditions and postures [9–12,14,15]. Briefly, 89 reflective markers were placed over the thorax from the clavicles to the anterior superior iliac spines along pre-defined vertical and horizontal lines. The detailed position of the markers is described in detail elsewhere [9]. The three-dimensional position of each marker was measured by an automatic motion analyzer (ELITE System, BTS, Milano, Italy) at a frequency of 10 Hz using four special video cameras, two in front and two behind the subject. Chest wall volume was determined by approximating the chest wall surface by triangles connecting the markers and computing the volume enclosed by all these triangles. The use of a subset of the markers allowed us to measure separately the volume of three chest wall compartments subdividing Vcw into the pulmonary rib cage volume (V_RCp_), defined as the part of the chest wall between the clavicular line and the horizontal line at the level of the xiphoid process, the abdominal rib cage volume (V_RCa_), defined as the volume comprised between the xiphoid process and the costal margin and the abdominal volume (V_AB_), defined as the volume comprised between the costal margin and the anterior superior iliac spine. This technique was proven to be reliable when used to measure total tidal volumes and all the other ventilatory parameters. Moreover, because it is based on the direct measurement of volumes, it can be used to track breath-to-breath changes in end-expiratory lung volume (EEV_L_)[12]. In particular, it has been shown that the tidal volume of the total chest wall measured by OEP is extremely similar to the tidal volume measured by spirometry, with the coefficient of variations of the differences between techniques being below 5% [9], Therefore we assume that all breathing pattern parameters derived by the total chest wall volume measured by OEP accurately resembles the ones measured by spirometry or body plethismography. In this study, from the time course of Vcw all classical breathing pattern parameters were computed. In particular, we considered the total and compartmental chest wall tidal volumes (V_T_cw, V_T_rc,p and V_T_ab), the total and compartmental chest wall end expiratory volumes (EEVcw, EEVrc,p, EEVab), the total respiratory time (T_TOT_) with its components for the inspiratory (T_I_) and expiratory (T_E_) phases, the duty cycle (T_I_/T_TOT_), the respiratory rate (RR) and the intra-breaths interval (IBI). The combination of these time-based parameters with volume changes allowed also the assessment of the minute ventilation (V_E_) and mean inspiratory flow (V_I_/ T_I_).

In order to assess the repeatability of the correlation properties of various parameters in the same subject, the measurements were repeated in a subset of 12 subjects from 3 to 9 months after the first measurement. The protocol was approved by the institutional review board of Fondazione Don Carlo Gnocchi, Milano and written informed consent was obtained from each subject.

### Data analysis

#### Long-range correlation properties by DFA

First, from the time courses of Vcw, all end-expirartory (EE) and end-inspiratory (EI) points were identified and used to compute all the breathing pattern parameters and end-expiratory volumes for each breath and to build, for each parameter, a time series as a function of breath number. These time series were analyzed by the DFA as suggested by Peng et al. [13]. The root-mean-square fluctuation of the integrated and detrended time series of *y*(*k*) of a time series was computed as follows:

$$F(n)={\sqrt{\frac{1}{N}{\sum_{k=1}^{N}\left(y(k)-y_{n}(k)\right)}^{2}}}^{}$$

To calculate F(*n*) the time series y(k) containing *N* data points, the original time series were first integrated:

$$y(k)=\sum_{i=1}^{k}\left(x(i)-\bar{x}\right)$$

where *x*(*i*) is the ith value of time series of one of the respiratory variables and $\bar{x}$ is the corresponding average of the time series. The time series *y*(*k*) was then divided into non-overlapping windows of equal length (*n*). A linear regression line was fit through the data points of *y*(*k*) in each window. The regression line *y*_*n*_(*k*) established the local trend in that window. The time series *y*(*k*) was then detrended by subtracting the local trend, *y*_*n*_(*k*), from the data in each window. The calculation of F(*n*) was repeated for different *n* and plotted as a function of *n* on a log-log plot. When F(*n*) shows a linear increase on the log-log plot, then F(*n*) is said to follow a power-law functional form:

$$F(n)=An^{\alpha }$$

where α is the scaling exponent and *A* is the amplitude of the power-law fluctuation function. These parameters can be obtained as the slope and intercept, respectively, of a straight line fit through the data plotted on a double logarithmic graph.

For a random process, α has the value of 0.5. For a positively correlated signal (large fluctuations are likely to be followed by large fluctuations), α is >0.5, and for an anticorrelated signal (large fluctuations are likely to be followed by small fluctuations), α is between 0 and 0.5 [13]. If F(n) follows a power law over at least an order of magnitude time scale with an α different from 0.5, the corresponding variable is said to exhibit long-range correlations or scale-invariant behaviour. For example, α = 1 corresponds to 1/f noise and α = 1.5 corresponds to Brownian noise.

To ascertain that the correlations in breath-to-breath fluctuations of the parameters are real, we randomized (shuffled) the order of the original breath-to-breath time series. Such a rearrangement of the data results in an uncorrelated time series. Thus, although this procedure does not alter the distribution of the amplitudes in the time series, the correlated ordering should disappear, and hence α of the shuffled time series should be 0.5, allowing to establish the existence of long-range correlations for a given variable if the value of α is significantly different from that after shuffling.

To assess the sensitivity of α to the number of breaths analysed (i.e. the length of the recording), we also estimated α by considering different numbers of breaths. We produced 5 different time series for each of the EEV recording by including all the breaths from the first to the 2^N^-th breath and computed α for each time series reporting α as a function of N.

#### Neuromechanical model of lung function fluctuations

In order to account for the correlated behavior of the data, we modified the model of Cernelc et al. [4], which was able to account for the observed long-range correlations in breath-to-breath fluctuations of V_T_, O_2_ and CO_2_ in babies. The model is based on the original neural oscillator model proposed by Botros and Bruce [8]. The neural oscillator consists of five coupled non-linear ordinary differential equations corresponding to the activities of five neuron groups in the brain respiratory centre (Fig. 1B). These neuron groups receive tonic inputs (TNI) from the periphery and other brain centres and the network, combined with the TNIs, generates a rhythmic output. Specifically, the ramp-inspiratory neuron group provides periodic outputs to the phrenic nerve which, in turn, activates the respiratory muscles. Additionally, we also implemented a feedback loop accounting for the pulmonary stretch receptors (Fig. 1B) that receive input from the ramp-inspiratory group and send excitatory and inhibitory signals to the late-inspiratory and early-inspiratory groups, respectively. Table 1 shows the parameters of the full model.

**Table 1:** Parameter values defining the respiratory brain oscillator consisting of 5 neuron groups and the pulmonary stretch receptors.

	R-I	L-I	P-I	Exp	E-I	PSR
**R-I**	0.7	1.361	0	−0.729	−1.8	4.0
**L-I**	−5.0	2.3	0.0	0.0	0.0	0.0
**P-I**	−1.719	−3.0	1.54	0.0	−2.15	0.0
**Exp**	−1.371	−0.793	−1.351	1.55	0.0	0.0
**E-I**	0.0	−2.056	−2.254	−2.254	0.65	0.0
**PSR**	0.0	1.1	0.0	0.0	−0.1	2.5

R-I: ramp-inspiratory; L-I: late-inspiratory; P-I: post-inspiratory; Exp: expiratory; E-I: early-inspiratory; PSR: pulmonary stretch receptors. The diagonals represent self-excitatory feedback to each neuron group. Positive and negative values are excitatory and inhibitory, respectively.

We first solved the differential equations of the network in the time domain using MATLAB (Mathworks Inc, Natick, MA, USA). Following a short transient, the solution of the network becomes strictly periodic without any irregularities. To mimic the observed irregularities, we added noise to the TNI of the first or ramp-inspiratory neuron group (TNI_1_), based on considerations of Hoop et al. [16] suggesting that neural noise varies within the respiratory cycle most likely due to varying chemoreceptor and stretch receptor responses. Hoop et al. [16] found correlations in the neural noise itself. However, in order to test whether the dynamic nonlinear oscillator alone is able to generate long-range correlations, we added uncorrelated random noise to the input of the oscillator. The mean value of TNI_1_ was 3 with a uniformly distributed noise between 1 and 5 (standard deviation, SD = 1.15) which changed on average 1.6 times within the respiratory cycle. Next, we simulated ~500 breaths by solving the 6 coupled nonlinear differential equations using MATLAB. The negative parts of the cycles corresponding to expiration were replaced by zeros, the signal was then resampled at equidistant time intervals and was further used to drive a mechanical model of the respiratory system.

The mechanical properties of the chest and respiratory system were modelled by using a Kelvin body which is a parallel combination of a spring (E_1_ = 1) with an in series combination of another spring (E_0_ = 1) and a dashpot (R_0_ = 40). This model has been used to describe the mechanics of the respiratory system [17,18]. The output of the ramp inspiratory group was assumed to provide a pressure which was used to drive the Kelvin body. The volume displacement of the Kelvin body, which corresponds to tidal volume, was then solved in the time domain in MATLAB for several combinations of the parameters. When the driving pressure increases during inspiration, the volume displacement also increases with a certain time lag due to the viscoelastic time constant of the Kelvin body. Once inspiration ends, the Kelvin body passively relaxes and the volume displacement decreases. Depending on the neural noise reaching the brain neural oscillator within the respiratory cycle, the oscillator can slow down or accelerate even within a cycle. If the inspiration starts before the Kelvin body reaches its fully relaxed state, the next inspiration becomes superimposed on the relaxation curve. Consequently, the absolute displacement of the Kelvin body, which mimics EEV_L_, also increases. Since the TNI_1_ shows irregular fluctuations, the end-expiratory lung volume should also display apparently random cycle-by-cycle fluctuations.

Because there was no feedback from the Kelvin body to the oscillator, we could simulate various long time series of the ramp-inspiratory group which were subsequently used as inputs to the Kelvin body. Since the solution for the displacement of the Kelvin body turned out to be highly sensitive to how the pressure input was sampled, we used a stiff differential equation solver. To test the numerical algorithm, first the noise in the neural network was set to zero which provided a strictly periodic output. This signal was used to drive the Kelvin body and the parameters of the solver were then adjusted until no variation was seen in the simulated EEV_L_. Noise was then added to the neural oscillator and the simulations were repeated for short and long time constants of the Kelvin body. This procedure allowed us to examine the correlation properties of the time series of T_TOT_, V_T_ as well as EEV_L_ using the DFA algorithm.

#### Statistical analysis

Data are reported as mean ± SD and were compared using one-way or two-way ANOVA. When the normality or equal variance tests failed, either non-parametric tests were used or the data were transformed before the parametric test. Significance was accepted at the level of p<0.05.

## Results

**Fig 2:**
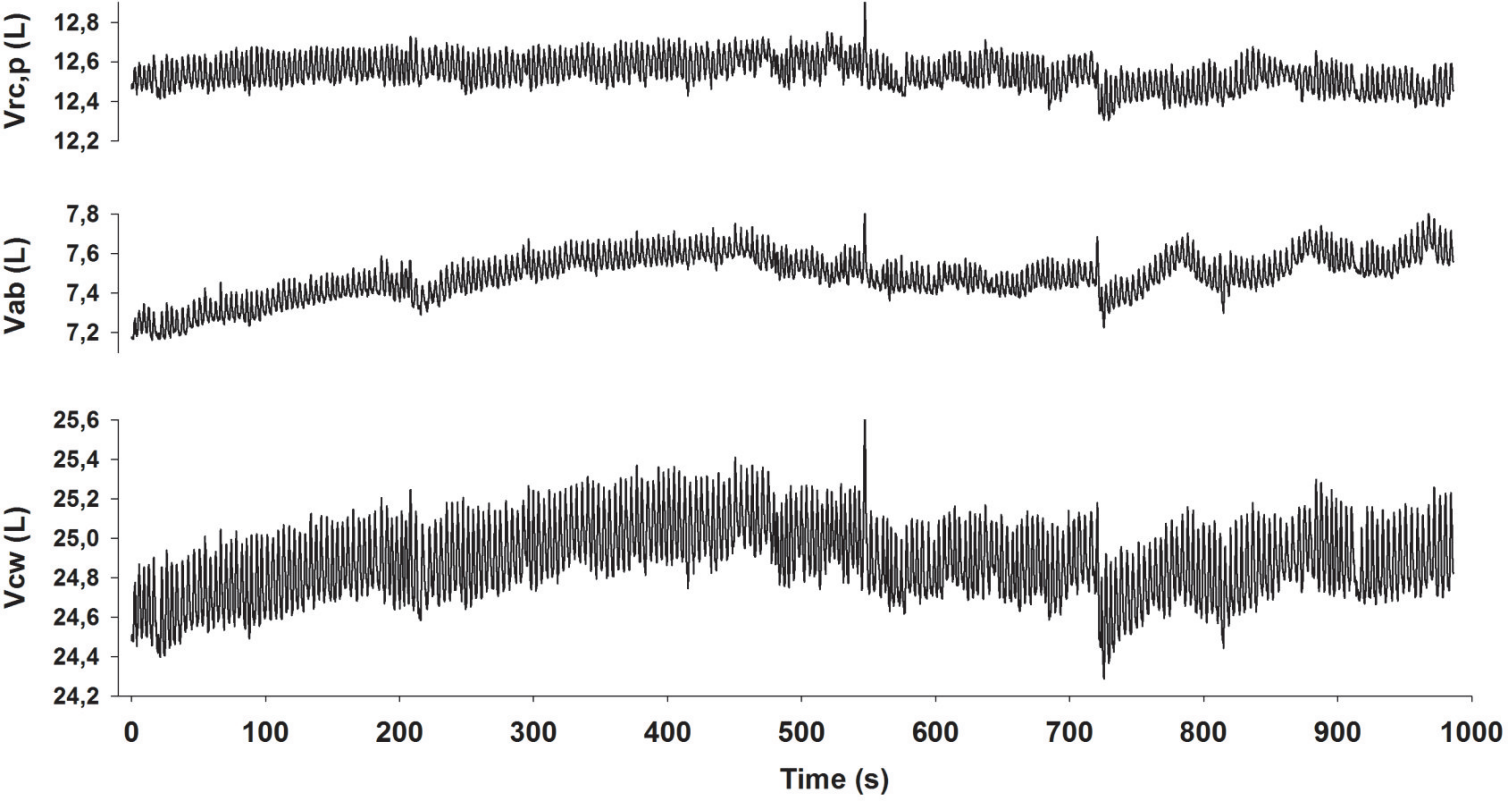
Experimental tracings of total and compartmental chest wall volumes recorded continuously on a representative subject by Opto-Electronic Plethysmography (only 16 min of tracings are reported for clarity). Vcw: total chest wall volume; Vab: abdominal volume; Vrc,p: volume of the pulmonary rib cage.

**Fig 3:**
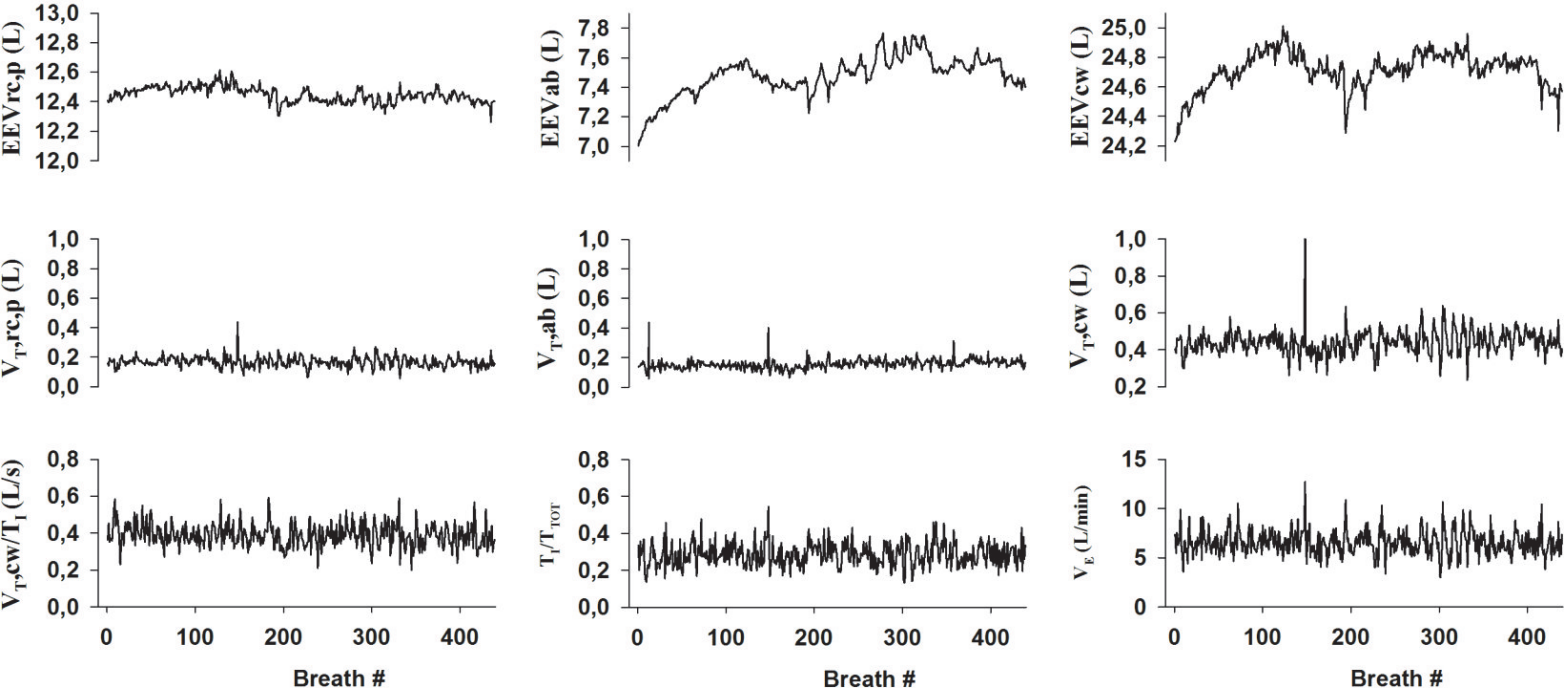
Time series of the different breathing patter parameters computed from the volume recordings on a representative subject.

For each subject, we recorded approximately 500 breaths. Fig. 2 shows examples of the time course of the total chest wall volume (Vcw) and its pulmonary rib cage (Vrc,p) and abdominal (Vab) components for a representative subject. It can be seen that the fluctuations in volume are noticeably larger in the abdominal than in the rib cage compartment. The time series of several ventilatory parameters were then obtained by detecting EE and EI points and computing the value of each parameter on a breath-by-breath basis (Fig. 3).

The end-expiratory volume of the abdominal compartment (EEVab) showed a significantly larger variability than that of the pulmonary rib cage (EEVrc,p). Since also the magnitude of the changes of EEVab was larger than for the EEVrc,p, the fluctuations in the end-expiratory volume of the total chest wall (EEVcw) were very mostly dominated by those in EEVab. The tidal volumes of the two chest wall compartments and the total chest wall as well as the inspiratory flow, duty cycle and the minute ventilation all showed qualitatively similar fluctuations although somewhat different from EEVab.

**Fig 4:**
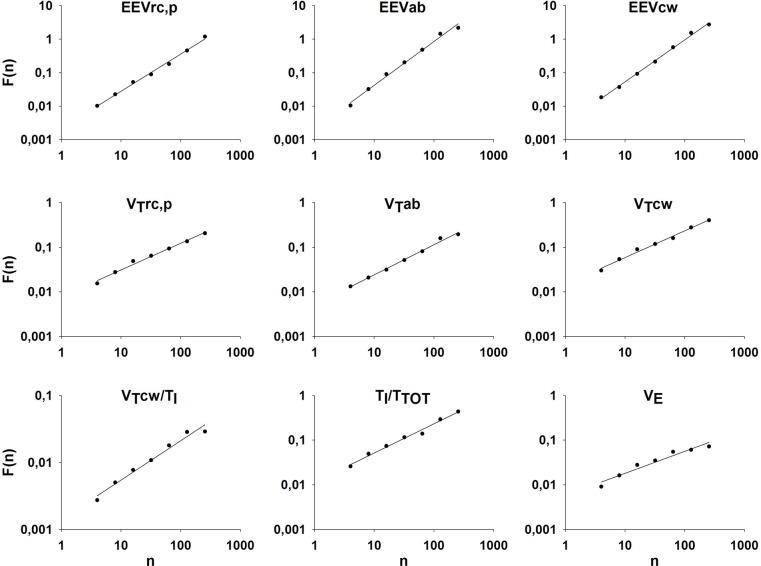
Fluctuation (F) as a function of window size (n) for the time series from volume tracings of a representative subject. Each panel refers to a specific variable of the breathing pattern. See text for abbreviations. The regression line determining α is also reported.

The DFA plots corresponding to the time series in Fig. 3 are summarized in Fig. 4. It can be seen that except for minute ventilation (V_E_), the fluctuation function of each ventilatory parameter in this subject increases linearly on a double logarithmic graph through about 2 decades of breath numbers. For this representative subject, the values of the exponent α obtained as the slope of the linear regression fits to the data were higher than 1 for all end-expiratory volumes. The values of α were between 0.58 and 0.65 for all other ventilatory parameters except V_E_ suggesting that EEV was considerably more correlated than the other parameters. With regard to V_E_, when all 7 points on the DFA plot were included in the regression, α was 0.49 (implying a random process) with an r^2^ = 0.972. However, it is clear that the last two points deviate from a straight line. By removing the last two points corresponding to the longest timescales from the regression, α became 0.63 with an r^2^ = 0.99. Regarding all the subjects, we observed a similar trend (i.e. r^2^<0.98) in V_E_ in 4 and in V_T_/Ti in 1 of the 16 subjects. The subject showing a low r^2^ for the DFA fit of V_T_/Ti was also one of the 4 subjects showing low r^2^ in V_E_. In these cases, we excluded the last 1 or 2 points from the linear regression to maximize r^2^. In 2 of the 4 subjects, r^2^ did not increase above 0.98 and hence the corresponding α values were excluded from the statistics.

Table 2 summarizes the mean±SD values of the average, the coefficient of variation (CV) and α of the ventilatory parameters EEVcw, EEVrc,p, EEVab, V_T_cw, V_T_rc,p and V_T_ab for all subjects. The CV of EEVab was statistically significantly larger than that of the EEVcw and EEVrc,p. The larger variability was also accompanied by stronger correlation properties of EEVab because the corresponding α was statistically significantly larger than the α of EEVcw or EEVrc,p. Interestingly, with regard to tidal volume, the largest variability was seen in the rib cage compartment which had a significantly larger CV than the total chest wall or the abdominal compartment. The largest α for the tidal volumes was observed in V_T_ab which reached statistical significance compared to the α of V_T_cw. When the experiments were repeated on a second day 3 to 9 months later, no differences were found between any of the parameters.

**Table 2:** 

	End Expiratory Volumes			Tidal Volumes		
	CW	RC,p	AB	CW	RC,p	AB
**Mean**	19.683 ± 3.719	11.566 ± 1.893	4.905 ± 1.154	0.516 ± 0.174	0.214 ± 0.079	0.202 ± 0.076
**CV**	0.0040 ± 0.0015^§^	0.0036 ± 0.0012^§^	0.0105 ± 0.0048* ^†^	0.303 ± 0.165^†^	0.388 ± 0.191* ^§^	0.283 ± 0.166^†^
**α**	1.064 ± 0.136^§^	1.046 ± 0.065^§^	1.125 ± 0.118* ^†^	0.669 ± 0.130^§^	0.709 ± 0.131	0.734 ± 0.125*

Statistical differences:

* vs. total chest wall (CW),

† vs. pulmonary rib cage (RC,p),

§ vs. abdomen (AB).

Table 3 summarizes the mean±SD values of the average, CV and α of additional ventilatory parameters related to the respiratory period. The T_I_/T_TOT_ showed considerably smaller variability than the other parameters. Indeed, the CV of T_I_/T_TOT_ was statistically significantly smaller than that of the other parameters. The value of α for T_TOT_ was significantly larger than α for T_I_/T_TOT_ or V_E_. Again, on the second day, the parameter values were statistically the same as on the first day suggesting a good repeatability of the measurements.

**Table 3:** 

	T_TOT_ (s)	V_T_/T_I_ (L/s)	T_I_/T_TOT_	V_E_ (L/min)
**Mean**	4.335 ± 1.049	0.321 ± 0.098	0.39 ± 0.035	7.326 ± 2.282
**CV**	0.241 ± 0.109^§^	0.27 ± 0.078^§^	0.179 ± 0.045* ^†^ ^‡^	0.254 ± 0.096^§^
**α**	0.702 ± 0.102^‡^	0.65 ± 0.095	0.618 ± 0.082	0.618 ± 0.120*

Statistical differences:

* vs. T_TOT_,

† vs. Vt/T_I_,

§ vs. T_I_/T_TOT_,

‡ vs. V_E_.

To summarize, all ventilatory parameters showed significant fluctuations and had a correlation exponent higher than 0.5, implying the presence of long-range correlations in their fluctuations. While the end-expiratory lung volumes had the smallest variability with respect to the average, they also showed the strongest correlations. In particular, the abdominal compartment had the largest exponent implying the strongest control on the fluctuations over long time scales. On the other hand, the least amount of correlation (smallest value of α) was seen in the T_I_/T_TOT_ while the parameters showing the largest coefficient of variation were the total and compartmental V_T_.

**Fig 5:**
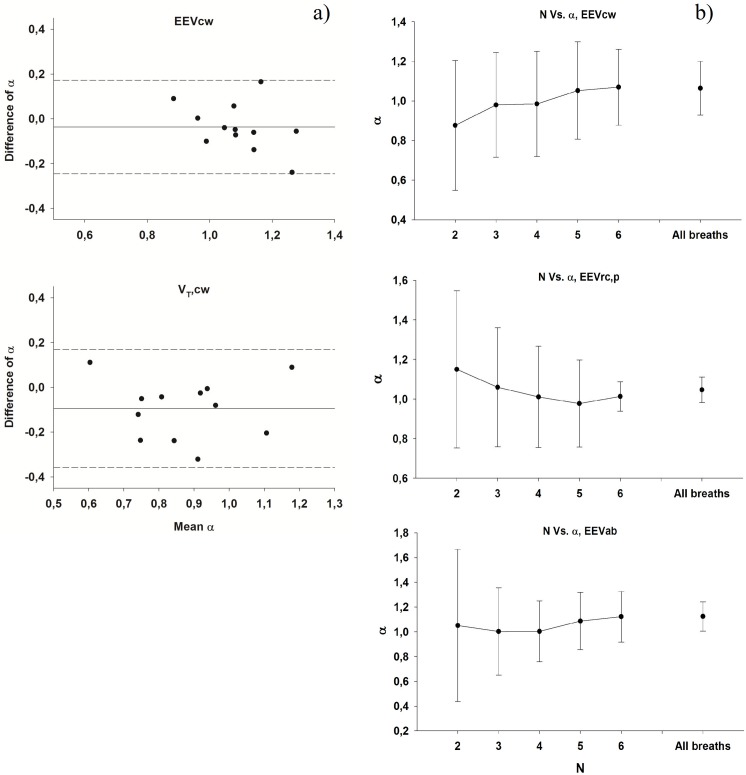
a) Bland-Altman analysis for the repeatability of the chest wall end-expiratory (EEVcw, upper panel) and tidal (V_T_,cw, lower panel) volumes long term correlations. Each data point represent the value of α measured in the same subject during two different experimental session with from 3 to 9 month time interval between the measurements. **b)** α values as a function of the number of breaths considered for the analysis for total and compartmental end-expiratory chest wall volumes.

The repeatability data are reported as Bland and Altman graphs in Fig. 5A. The average difference in α for the EEV_L_ time series is 0.037±0.107, with no evident dependence of the difference on the mean value. The sensitivity of α as a function of the length of the recording is also shown in Fig. 5B. The results demonstrate that α approaches a stable value already for time series of length 64.

**Fig 6:**
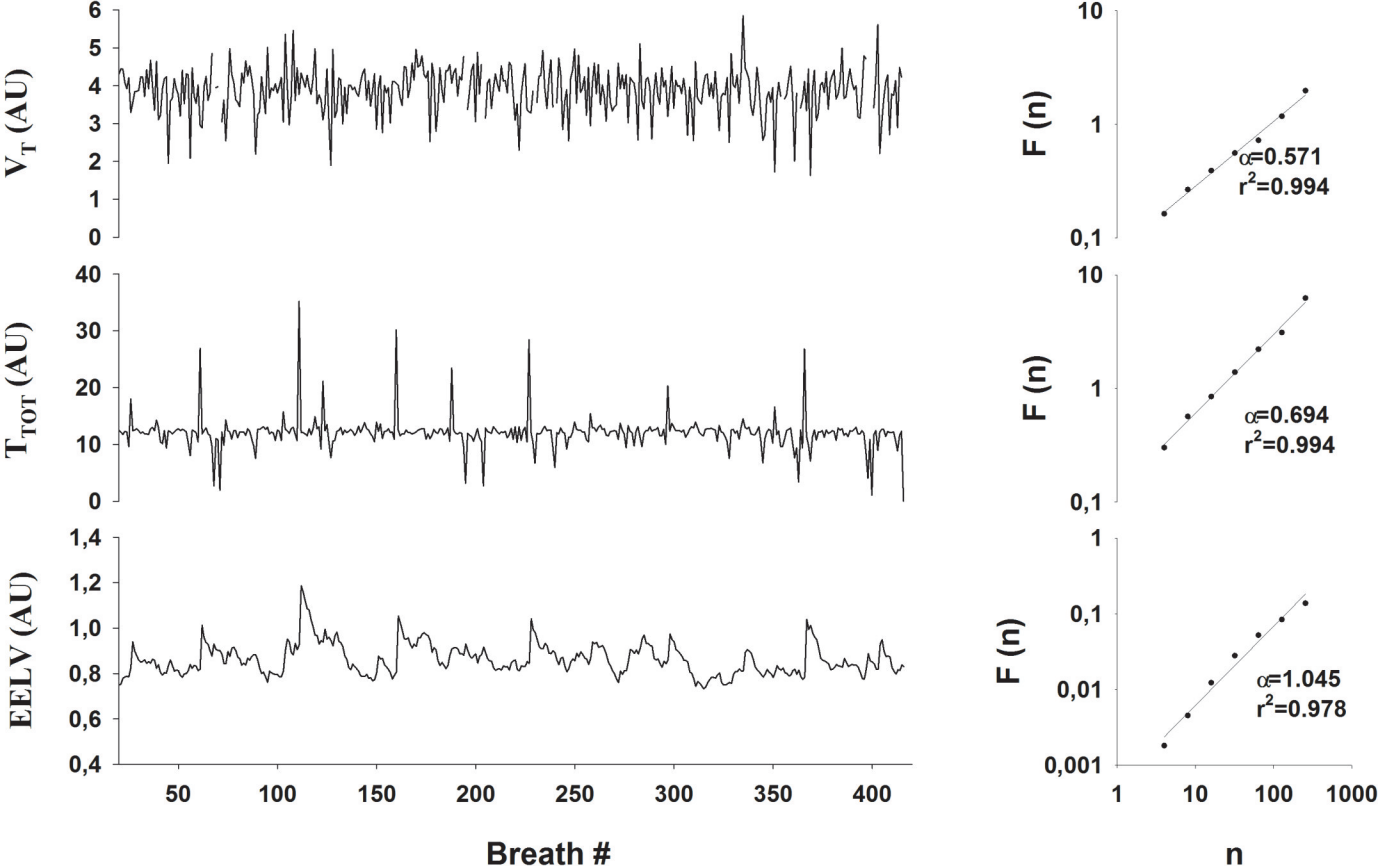
Left panels: time series derived from the simulated volume tracings provided by the model for tidal volume (V_T_, upper panel), respiratory time (T_TOT_, middle panel) and end-expiratory lung volume (EELV, lower panel). Right panels: Fluctuation (F) as a function of window size (n) for the time series from volume tracings simulated by the model (V_T_, upper panel, T_TOT_, middle panel, EELV, lower panel).

In order to better understand the origins of correlation properties of the breath-by-breath fluctuations in respiratory parameters, we simulated the fluctuations in V_T_, T_TOT_, and EEV_L_ using the Kelvin body driven by the respiratory neural oscillator network model. Examples of the corresponding time series are shown in Fig. 6A. Fluctuations in V_T_ of the model are most similar to those in V_T_cw (Fig. 3), whereas those in T_TOT_ and EEV_L_ are somewhat reminiscent of the corresponding fluctuations in the abdominal compartment. Fig. 6B shows the fluctuation functions for the model outputs on double logarithmic graphs. The model-based α for V_T_ is 0.571 overlapping with the experimental values of α for the chest wall (0.669 ± 0.13). The model correctly predicted both the α for T_TOT_ (0.694 vs 0.702 ± 0.1) and the α for EEV_L_ (1.045 vs 1.064 ± 0.136).

## Discussion

The most important finding of this study is that the long-term correlation properties of EEV parameters are significantly different (greater) than those of all other respiratory indices such as V_T_ or T_TOT_.

Even though several studies investigated the correlation properties of breathing pattern[2–5,7], only few addressed the correlation properties of EEV such as functional residual capacity (FRC) in humans, likely because of the difficulties in continuous monitoring. To our knowledge, the only study addressing this issue is by Hlastala et al. [19], in which the authors used a complicated set-up based on total body plethysmography in supine position to record FRC fluctuations and other respiratory parameters in four healthy subjects for 40 min. The experimental data reported, i.e. representative tracings of time series of breathing pattern parameters and FRC are quite similar to ours, showing much greater long time scale fluctuations for FRC compared to V_T_ and other respiratory parameters. In that study, however, the analysis of the fluctuations was limited to autocorrelation and spectral analysis, which showed major oscillations of FRC with periods from 8.3 and 28 min with amplitudes varying from 42 to 176 ml.

In this study, the use of OEP allowed us to record accurate time series of FRC in a way that minimally perturbed natural breathing, as this technique does not require any connection to the airway opening and, therefore, no external load or dead space is added to the subject’s respiratory system. The accuracy of this technique for the long-term measurement of changes in EEV has been validated [12], showing the absence of any significant drift on long time scales. Therefore, the data presented in this study accurately reproduces natural variations in EEVs in conscious humans.

The long term correlations of EEV_L_ were studied by Zhang et al. [20] in anaesthetised tracheotomised rats exposed to either constant positive or negative airway pressure using the Hurst exponent (H). They found a value of 0.55 for H when positive airway pressure was applied which statistically significantly increased to 0.64 when negative airway pressure was applied. Interestingly, the difference in H between the positive and negative pressure conditions vanished if the rats were vagotomised, with H approaching 0.55 in all conditions. The authors concluded that although the exact mechanisms for the fractal behavior of EEV regulation are unknown, the results suggest that interaction of the pulmonary mechanoreceptor activity with the central respiratory pattern generator might contribute to the observed long-term correlation. Furthermore, in a subsequent study [21], the same authors found similar H values in rats with intact airways, supporting their previous conclusion. Since H is equal to α for fractional Gaussian noise, the nature of the long term correlations in anaesthetised rats appears to be much less correlated than in conscious humans in the present study. There are several possible reasons for the difference. For example, correlation properties of V_T_ and T_i_ have been shown to depend on sleep stage [5]. Additionally, the α of respiratory resistance at 8 Hz in human subjects decreased from wake to sleep [5] suggesting less active brain control of respiratory variables during sleep.

In order to interpret our experimental results, we extended the neural network model of Cernelc et al. [4] to include pulmonary stretch receptor feedback and the mechanical load of the respiratory system on the respiratory muscles. Without the pulmonary stretch receptor feedback (Fig. 1B), the model could predict α for V_T_ and EEV_L_, but it provided a lower value for T_TOT_ (α = 0.534). With regard to the mechanical load, we hypothesized that while timing of the respiratory cycle and V_T_ are mostly determined by the respiratory oscillator and the inputs to the oscillator, EEV_L_ volume would also be influenced by the passive mechanical properties of the tissues. The reason is that, although some expiratory muscles are active during expiration and may also be active during inspiration for smoothing the shape of tidal volume or maintaining the posture [22], changes in lung volume are mostly determined by the passive mechanical properties of the respiratory tissues. While the lack of active expiration in the model is a limitation, the simulations are in reasonable agreement with the measured data. Thus, in the absence of active expiration, the tissues are stretched at end-inspiration and the stored elastic energy in the tissues lower lung volume via relaxation. Because the tissues are viscoelastic, this is similar to the discharge of a capacitor through a resistor. Thus, we used the simplest viscoelastic model, the Kelvin body, to mimic both inspiration and expiration. During inspiration, the Kelvin body simply acts as a filter and slightly shapes the inspiratory volume and therefore it has little effects on the correlations of V_T_ and T_TOT_. Indeed, simulations without the Kelvin body changed α for both variables by less than 1.5%. Since during expiration the oscillator input to the Kelvin body is zero, the effect of the load on the α of EEV_L_ is substantial.

The assumptions used in and the limitations of the neural network model itself have been discussed in previous studies [4,7] and hence we focus here on the limitations of the mechanical model of the respiratory system. This model is a simple Kelvin body that has been used to fit the mechanical properties of the respiratory system in healthy human subjects [17]. The Kelvin body is a simple linear viscoelastic model with a dashpot and two springs. The dashpot with a spring in series mimics the viscoelastic properties of the tissues whereas the parallel spring gives the model a solid-like behaviour: following a step change in force (or pressure), the displacement (or volume) approaches a finite constant during creep and the time course of the creep is characterized by a single exponential [23]. However, frequency domain measurements of the respiratory tissues including the chest wall and the lung tissues have unequivocally proved that the tissues have a broad spectrum of time constants and this has led to the concept of the so called “constant phase” model [24]. The single exponential of the Kelvin body cannot account for the broad distribution of time constants of real tissues and hence the viscoelastic memory of this model can only describe tissue behaviour over a limited time scale. The constant phase model has a power law type of time constant distribution and its memory, characterized by the relaxation of the creep function, also has a power law form [25]. Thus, white noise passing through such a system generates long-range correlations and the corresponding DFA plot is a straight line over many decades of time on a double logarithmic graph. As seen in Fig. 5, however, the DFA plot for the EEV_L_ obtained from our model is curved because the memory function of the Kelvin body does not follow a power law and this is a limitation of the model. Indeed, for short time scale, a local α = 1.4 fits the first 4 points of simulated EEV_L_ time series whereas for long time scales a weaker correlation is seen with α = 0.7 as a best fit for the last 4 points. Furthermore, sensitivity analysis also showed that the longer the time constant of the Kelvin body, the higher the α of the simulated EEV_L_. For example, a 10 times smaller time constant lowered α of the simulated EEV_L_ to 0.7.

Another limitation of the model is that the Kelvin body is linear, whereas the respiratory tissues exhibit non-linear mechanical properties [26]. An advantage of the Kelvin body model is that it is easy to simulate force-displacement data in the time domain with or without nonlinearities. Therefore, we tested the effects of mild nonlinearities on the output of the Kelvin body. The nonlinearity was an additional contribution (F_n_) to the total force output of the Kelvin body in the form of a second order term proportional to the square of the displacement: F_n_ = E_2_*x^2^ where E_2_ is the nonlinear spring and x is the displacement of the Kelvin body. We found that with E_2_/E_1_<0.2 there was no appreciable effect of nonlinearity on the simulated time series. For E_2_/E_1_ = 0.5, α of the simulated EEV_L_ decreased by 15% but the exponent of T_TOT_ and V_T_ did not change. Since the time constant R_0_/E_0_ and the nonlinearity had opposing effects on α of EEV_L_, it was not possible to uniquely fit the Kelvin body to measured data. However, the purpose of the modelling was not to estimate parameters of individual subjects; rather, to examine whether the neural network together with a mechanical load is capable of qualitatively accounting for the measured correlations.

Despite the simplicity of the model, it provided correlated fluctuations with exponents that are in reasonable agreement with the experimentally measured exponents. These correlated fluctuations come from the brain but the input from the periphery is crucial for maintaining the oscillations [8]. On the other hand, without fluctuations in the inputs to the neural oscillator, the output of the oscillator would be a deterministic cyclic pattern, a limit cycle, with constant frequency and amplitude. One interesting implication of the modelling results is that the addition of the Kelvin body did not significantly affect the pattern including timing (T_TOT_). This suggests that while the correlated properties of EEV_L_ are determined both by the brain oscillator and the viscoelastic memory of the tissues, breathing patterns originates almost exclusively from the brain and the neural noise.

There is another important implication of the fluctuations in V_T_ for cell function. Since the oscillator is a nonlinear network of neuron groups, any fluctuation coming from outside—including the lung and other brain regions—will generate cycle-by-cycle variations both in the rate and amplitude of the output which is assumed to be the force generated by the respiratory muscles. Consequently, all cells in the respiratory system are exposed to cycle-by-cycle fluctuations in stretch due to the variability in V_T_. Since such fluctuations must have been present for hundreds of millions of years when the earliest mammals evolved, cells in the respiratory system must have adapted to such variabilities in stretch. One example of this is that surfactant secretion by alveolar type II epithelial cells is sensitive to stretch pattern and cells receiving variability in stretch secrete more surfactant both in vivo [27] and in vitro [28]. Therefore, we conclude that the origin of correlated fluctuations observed in breathing parameters is the brain respiratory center that receives inputs from other brain centers and the periphery. Furthermore, the cycle-by-cycle fluctuations in tidal volume and respiratory times likely have a significant impact on general functioning of stretch-sensitive adherent cells in the respiratory system.

In summary, we measured the cycle-by-cycle variations of several respiratory physiological variables and found that these fluctuations show long-range correlations that are highly reproducible in individual subjects. Using a novel neuromechanical model, we propose that the correlations in the timing and amplitude of the physiological variables originate from the brain with the exception of end-expiratory lung volume which shows the strongest correlations due to the contribution of the viscoelastic properties of the tissues. Finally, we also suggest that since cells in the respiratory system are exposed to fluctuation in cycle-by-cycle stretch related to variability in tidal volume, these finding may have implications on general cell function in the respiratory system.

## References

[1] Glass L (2001) Synchronization and rhythmic processes in physiology. Nature 410: 277–284. Available: http://www.ncbi.nlm.nih.gov/pubmed/11258383. Accessed 15 October 2013. 11258383 10.1038/35065745

[2] Fadel PJ, Barman SM, Phillips SW, Gebber GL (2004) Fractal fluctuations in human respiration. J Appl Physiol 97: 2056–2064. Available: http://www.ncbi.nlm.nih.gov/pubmed/15286051. Accessed 22 October 2013. 15286051 10.1152/japplphysiol.00657.2004

[3] Peng CK, Mietus JE, Liu Y, Lee C, Hausdorff JM, et al (2002) Quantifying fractal dynamics of human respiration: age and gender effects. Ann Biomed Eng 30: 683–692. Available: http://www.ncbi.nlm.nih.gov/pubmed/12108842. Accessed 22 October 2013. 12108842 10.1114/1.1481053

[4] Cernelc M, Suki B, Reinmann B, Hall GL, Frey U (2002) Correlation properties of tidal volume and end-tidal O2 and CO2 concentrations in healthy infants. J Appl Physiol 92: 1817–1827. Available: http://www.ncbi.nlm.nih.gov/pubmed/11960929. Accessed 15 October 2013. 11960929 10.1152/japplphysiol.00675.2001

[5] Campana LM, Owens RL, Butler JP, Suki B, Malhotra A (2013) Variability of respiratory mechanics during sleep in overweight and obese subjects with and without asthma. Respir Physiol Neurobiol 186: 290–295. Available: http://www.ncbi.nlm.nih.gov/pubmed/23473922. Accessed 15 October 2013. doi: 10.1016/j.resp.2013.02.026 23473922 10.1016/j.resp.2013.02.026 PMC3659817

[6] Brack T, Jubran A, Tobin MJ (2002) Dyspnea and decreased variability of breathing in patients with restrictive lung disease. Am J Respir Crit Care Med 165: 1260–1264. Available: http://www.ncbi.nlm.nih.gov/pubmed/11991875. Accessed 24 September 2013. 11991875 10.1164/rccm.2201018

[7] Frey U, Silverman M, Barabási AL, Suki B (1998) Irregularities and power law distributions in the breathing pattern in preterm and term infants. J Appl Physiol 85: 789–797. Available: http://www.ncbi.nlm.nih.gov/pubmed/9729549. Accessed 15 October 2013. 9729549 10.1152/jappl.1998.85.3.789

[8] Botros SM, Bruce EN (1990) Neural network implementation of a three-phase model of respiratory rhythm generation. Biol Cybern 63: 143–153. Available: http://www.ncbi.nlm.nih.gov/pubmed/2375940. Accessed 15 October 2013. 2375940 10.1007/BF00203037

[9] Cala SJ, Kenyon CM, Ferrigno G, Carnevali P, Aliverti A, et al (1996) Chest wall and lung volume estimation by optical reflectance motion analysis. J Appl Physiol 81: 2680–2689. Available: http://www.ncbi.nlm.nih.gov/pubmed/9018522. Accessed 15 October 2013. 9018522 10.1152/jappl.1996.81.6.2680

[10] Aliverti A, Dellacà R, Pelosi P, Chiumello D, Gatihnoni L, et al (2001) Compartmental analysis of breathing in the supine and prone positions by optoelectronic plethysmography. Ann Biomed Eng 29: 60–70. Available: http://www.ncbi.nlm.nih.gov/pubmed/11219508. Accessed 15 October 2013. 11219508 10.1114/1.1332084

[11] Aliverti A, Dellacá R, Pelosi P, Chiumello D, Pedotti A, et al (2000) Optoelectronic plethysmography in intensive care patients. Am J Respir Crit Care Med 161: 1546–1552. Available: http://www.ncbi.nlm.nih.gov/pubmed/10806152. Accessed 15 October 2013. 10806152 10.1164/ajrccm.161.5.9903024

[12] Dellacà RL, Aliverti A, Pelosi P, Carlesso E, Chiumello D, et al (2001) Estimation of end-expiratory lung volume variations by optoelectronic plethysmography. Crit Care Med 29: 1807–1811. Available: http://www.ncbi.nlm.nih.gov/pubmed/11546992. Accessed 15 October 2013. 11546992 10.1097/00003246-200109000-00026

[13] Peng CK, Havlin S, Stanley HE, Goldberger AL (1995) Quantification of scaling exponents and crossover phenomena in nonstationary heartbeat time series. Chaos 5: 82–87. Available: http://www.ncbi.nlm.nih.gov/pubmed/11538314. Accessed 29 September 2013. 11538314 10.1063/1.166141

[14] Kenyon CM, Cala SJ, Yan S, Aliverti A, Scano G, et al (1997) Rib cage mechanics during quiet breathing and exercise in humans. J Appl Physiol 83: 1242–1255. Available: http://www.ncbi.nlm.nih.gov/pubmed/9338434. Accessed 15 October 2013. 9338434 10.1152/jappl.1997.83.4.1242

[15] Aliverti A, Cala SJ, Duranti R, Ferrigno G, Kenyon CM, et al (1997) Human respiratory muscle actions and control during exercise. J Appl Physiol 83: 1256–1269. Available: http://www.ncbi.nlm.nih.gov/pubmed/9338435. Accessed 15 October 2013. 9338435 10.1152/jappl.1997.83.4.1256

[16] Hoop B, Burton MD, Kazemi H, Liebovitch LS (1995) Correlation in stimulated respiratory neural noise. Chaos 5: 609–612. Available: http://www.ncbi.nlm.nih.gov/pubmed/12780216. Accessed 15 October 2013. 12780216 10.1063/1.166130

[17] Navajas D, Farré R, Canet J, Rotger M, Sanchis J (1990) Respiratory input impedance in anesthetized paralyzed patients. J Appl Physiol 69: 1372–1379. Available: http://www.ncbi.nlm.nih.gov/pubmed/2262456. Accessed 15 October 2013. 2262456 10.1152/jappl.1990.69.4.1372

[18] Suki B (1993) Nonlinear phenomena in respiratory mechanical measurements. J Appl Physiol 74: 2574–2584. Available: http://www.ncbi.nlm.nih.gov/pubmed/8335594. Accessed 15 October 2013. 8335594 10.1152/jappl.1993.74.5.2574

[19] Hlastala MP, Wranne B, Lenfant CJ (1973) Cyclical variations in FRC and other respiratory variables in resting man. J Appl Physiol 34: 670–676. Available: http://www.ncbi.nlm.nih.gov/pubmed/4703744. Accessed 15 October 2013. 4703744 10.1152/jappl.1973.34.5.670

[20] Zhang X, Bruce EN (2000) Correlation structure of end-expiratory lung volume in anesthetized rats with intact upper airway. Am J Physiol Regul Integr Comp Physiol 278: R1446–52. Available: http://www.ncbi.nlm.nih.gov/pubmed/10848510. 10848510 10.1152/ajpregu.2000.278.6.R1446

[21] Zhang X, Bruce EN (2000) Fractal characteristics of end-expiratory lung volume in anesthetized rats. Ann Biomed Eng 28: 94–101. Available: http://www.ncbi.nlm.nih.gov/pubmed/10645792. 10645792 10.1114/1.257

[22] Iscoe S (1998) Control of abdominal muscles. Prog Neurobiol 56: 433–506. Available: http://www.ncbi.nlm.nih.gov/pubmed/9775401. Accessed 15 October 2013. 9775401 10.1016/s0301-0082(98)00046-x

[23] Fung Y (1993) Biomechanics: Mechanical Properties of Living Tissues. Ney York: Springer-Verlag.

[24] Hantos Z, Daróczy B, Suki B, Nagy S, Fredberg JJ (1992) Input impedance and peripheral inhomogeneity of dog lungs. J Appl Physiol 72: 168–178. Available: http://www.ncbi.nlm.nih.gov/pubmed/1537711. Accessed 15 October 2013. 1537711 10.1152/jappl.1992.72.1.168

[25] Suki B, Barabási AL, Lutchen KR (1994) Lung tissue viscoelasticity: a mathematical framework and its molecular basis. J Appl Physiol 76: 2749–2759. Available: http://www.ncbi.nlm.nih.gov/pubmed/7928910. Accessed 15 October 2013. 7928910 10.1152/jappl.1994.76.6.2749

[26] Barnas GM, Sprung J (1993) Effects of mean airway pressure and tidal volume on lung and chest wall mechanics in the dog. J Appl Physiol 74: 2286–2293. Available: http://www.ncbi.nlm.nih.gov/pubmed/8335558. Accessed 15 October 2013. 8335558 10.1152/jappl.1993.74.5.2286

[27] Arold SP, Suki B, Alencar AM, Lutchen KR, Ingenito EP (2003) Variable ventilation induces endogenous surfactant release in normal guinea pigs. Am J Physiol Lung Cell Mol Physiol 285: L370–5. Available: http://www.ncbi.nlm.nih.gov/pubmed/12851212. Accessed 15 October 2013. 12851212 10.1152/ajplung.00036.2003

[28] Arold SP, Bartolák-Suki E, Suki B (2009) Variable stretch pattern enhances surfactant secretion in alveolar type II cells in culture. Am J Physiol Lung Cell Mol Physiol 296: L574–81. Available: http://www.pubmedcentral.nih.gov/articlerender.fcgi?artid=2670764&tool=pmcentrez&rendertype=abstract. Accessed 15 October 2013. doi: 10.1152/ajplung.90454.2008 19136581 10.1152/ajplung.90454.2008 PMC2670764

